# TLR4 and TLR7/8 Adjuvant Combinations Generate Different Vaccine Antigen-Specific Immune Outcomes in Minipigs when Administered via the ID or IN Routes

**DOI:** 10.1371/journal.pone.0148984

**Published:** 2016-02-10

**Authors:** Paul F. McKay, Deborah F. L. King, Jamie F. S. Mann, Guillermo Barinaga, Darrick Carter, Robin J. Shattock

**Affiliations:** 1 Imperial College London, Department of Infectious Diseases, Division of Medicine, Norfolk Place, London, W2 1PG, United Kingdom; 2 Infectious Disease Research Institute, Seattle, WA, 98102, United States of America; The Ohio State University, UNITED STATES

## Abstract

The induction of high levels of systemic and mucosal humoral immunity is a key goal for many prophylactic vaccines. However, adjuvant strategies developed in mice have often performed poorly in the clinic. Due to their closer similarity to humans, minipigs may provide a more accurate picture of adjuvant performance. Based on their complementary signalling pathways, we assessed humoral immune responses to model antigens after co-administration with the toll-like receptor 4 (TLR4) stimulator glucopyranosyl lipid adjuvant (GLA-AF) or the TLR7/8 agonist resiquimod (R848) (alone and in combination) via the intradermal (ID), intranasal (IN) or combined routes in the Gottingen minipig animal model. Surprisingly, we discovered that while GLA-AF additively enhanced the adjuvant effect of R848 when injected ID, it abrogated the adjuvant activity of R848 after IN inoculation. We then performed a route comparison study using a CN54 gp140 HIV Envelope model antigen adjuvanted with R848 + GLA-AF (ID) or R848 alone (IN). Animals receiving priming inoculations via one route were then boosted by the alternate route. Although differences were observed in the priming phase (IN or ID), responses converged upon boosting by the alternative route with no observable impact resultant from the order of administration (ID/IN vs IN/ID). Specific IgG responses were measured at a distal mucosal site (vaginal), although there was no evidence of mucosal linkage as these closely reflected serum antibody levels. These data indicate that the complex *in vivo* cross-talk between innate pathways are likely tissue specific and cannot be predicted by simple *in vitro* models.

## Introduction

Development of new adjuvants for mucosal and parenteral vaccination remains a key research priority for modern vaccinology [1]. This may be particularly important to the development of an effective HIV-1 vaccine where one of the greatest challenges is the elicitation of antibodies with sufficient breadth and potency to prevent viral acquisition at the mucosal portals of infection. In this study we evaluate the potential of two TLR agonists, selected on the basis of potential signaling cross-talk to promote systemic and mucosal response to a model HIV glycoprotein immunogen using a minipig model thought to better represent human responses than rodent species.

TLR agonists have a clear role as molecular components of vaccine adjuvants due to their ability to directly activate antigen-presenting cells (APCs) and enhance both humoral and cellular immune responses. Although TLRs as a group appear to have a certain degree of functional redundancy, each individual TLR, due to cellular location, interactions with cell surface or intracellular accessory molecules, and tissue-specific expression have the capacity to distinguish a wide range of pathogen signature molecular patterns [2]. TLRs can also be broadly grouped according to their dependence or independence on the adaptor molecule MyD88 [3]. Co-stimulation of these different pathways has the potential to stimulate complementary or synergistic effects, while antagonism more commonly occurs with agonists that act through the same pathway [4]. These attributes can be utilized by vaccinologists to tailor vaccine adjuvants to promote a particular immune response.

In this study we chose to investigate potential adjuvant effects of a combination of the synthetic monophosphoryl lipid A (MPLA) based TLR4 agonist, Glucopyranosyl Lipid Adjuvant (GLA), that acts in a TRIF pathway biased manner [5–7] and resiquimod (R848), a TLR7/8 agonist acting through MyD88 dependent signalling [4]. A number of previous *in vitro* studies using human APC, and in particular monocyte-derived macrophages and dendritic cells, have demonstrated synergy between TLR4 and TLR7/8 stimulation with enhanced cytokine production, reciprocal upregulation of each receptor [8, 9], and enhanced potential for activation of T-helper cell type 1 and/ or 17 responses [10–12]. The latter is effective in providing B cell help, promoting antibody production and class switch recombination [13, 14]. These data suggest amplified APC function in response to MYD88-TRIF cross-talk could enhance the induction of the immune response to a given vaccine *in vivo*.

We performed a series of iterative experiments to examine the inter-relationship of TLR4 and TLR7/8 agonist molecules, GLA-AF (an aqueous formulation of synthetic MPLA) and R848, on antigen-specific humoral outcome after vaccination via the IN and ID routes. We used the Göttingen minipig animal model as their immune system responses, particularly in respect of TLR ligand expression and stimulation, are thought to be more similar to humans than rodents [15–17]. To maximise the numbers of conditions screened whilst reducing animal usage we adopted a matrix experimental design using a range of model antigens. Subsequently we determined the impact of heterologous route prime-boost strategies on the induction of systemic and mucosal humoral immune response to a model HIV envelope glycoprotein vaccine (CN54gp140). Our data indicate that while addition of GLA-AF enhances adjuvantion of R848 when administered ID, it completely suppressed the adjuvant properties of R848 when administered IN. This study highlights the potential utility of the minipig model in the preclinical assessment of novel adjuvants designed for parenteral and nasal vaccination strategies.

## Materials and Methods

### Ethics Statement

The animal studies were approved by the Ethical Review Board of St. George’s, University of London where the experiments were carried out and work was performed in strict compliance with project and personal animal experimentation licences granted by the UK government in accordance with the Animals in Scientific Procedures Act (1986). Animals received minimal handling and their physical condition was monitored at least twice daily. All procedures were performed under isoflourane anesthesia when appropriate, and all efforts were made to minimize suffering. All animals enjoyed excellent health for the duration of the experiment and none became severely ill or died at anytime prior to the experimental endpoint. There was a detailed protocol in place, as per requirement of the humane endpoints described in the animal licence, for early euthanasia in the event of onset of illness or significant deterioration in condition. For mini-pigs the humane endpoints included; loss of appetite sufficient to lead to weight loss—the animals were monitored for weight daily, loss of movement, sedentary state, calls of distress indicating pain or discomfort, bruising at site of blood withdrawal, excessive or uncontrolled bleeding from site of blood withdrawal, incontinence, breathing difficulty, infection or necrosis at site of sampling (snout and vaginal). The presence of one of these indicators would lead to an assessment by a veterinary surgeon and the further welfare of the animals would be directed by them. In the case of an emergency if an animal became seriously ill or injured at any point when they were on the designated premises then the animal would be first stunned by captive bolt and then killed by exsanguination before the animal regains consciousness (a non-schedule 1 method). If it was possible to handle the animal without causing it further stress and/or injury to it or staff then a schedule 1 method would be used. The captive bolt would be administered by a person licensed to use a captive bolt. At the end of the experiment all animals were culled using a schedule 1 method and death confirmed before necropsy. Food and water were supplied ad libitum.

### Recombinant Proteins, GLA-AF and R848

The proteins used in this study were purified formulations of native proteins or recombinant proteins expressed in either prokaryotic or eukaryotic systems. Keyhole limpet haemocyanin (KLH) was separated from the limpet haemolymph by ammonium sulphate precipitation and further purified by chromatographic separation (Sigma, UK). Ovalbumin was directly purified from chicken egg whites and supplied as a lyophilized powder (Sigma, UK). Tetanus toxin was isolated from *Clostridium tetani* then inactivated by formaldehyde treatment and the toxoid derivative purified from solution by ammonium sulphate precipitation and resuspension in PBS (Pfenex Inc, USA). Recombinant HIV nef, produced in *E*. *coli*, was obtained from the Programme EVA Centre for AIDS reagents (NIBSC, UK and Diatheva, Italy) as a soluble protein in a 10% glycerol aqueous buffer. Beta-galactosidase was produced in *E*. *coli* and reconstituted from lyophilized powder (Sigma, UK). Recombinant *M*. *tuberculosis* early secreted antigenic target-6 kDa (ESAT-6) protein was produced in *E*. *coli* and purified by ion affinity and UF concentration solvent extraction (ImmunoDX, LLC, USA). Recombinant *M*. *tuberculosis* culture filtrate protein-10 kDa (CFP10) protein was produced in *E*. *coli* and purified by ion affinity, solvent extraction and UF concentration (ImmunoDX, LLC, USA). The hemagglutinin (HA) antigens were components of the Fluzone vaccine (Sanofi Pasteur, France) and contained HA from the 2011–2012 influenza season; A/California/07/2009 X-179A (H1N1), A/Victoria/210/2009 X-187 (H3N2) and B/Brisbane/60/2008. The HA proteins were separated from the virus by non-ionic surfactant (Triton® X-100) disruption of the formaldehyde inactivated influenza virions, producing a ‘split virus’ from which the HA proteins are further purified and then resuspended in PBS. HIV gp140, a trimeric gp140 clade C envelope (gp120 plus the external domain (ED) of gp41) and designated CN54gp140, was produced as a recombinant product in CHO cells and the protein manufactured to GMP specification by Polymun Scientific (Vienna, Austria). The identity of the product was confirmed by mass spectrometric analysis of tryptic fragments by the Medical Biomics Centre at St. George’s, University of London. The trimeric product was stable, and has been extensively tested to validate stability even when kept at room temperature (D. Katinger—personal communication) and has previously been reported to be immunogenic. All proteins were sourced from suppliers who were able to provide information on endotoxin levels and confirmed that the proteins were endotoxin low/free. We further tested the LPS endotoxin level of the material we used to vaccinate the pigs using a chromagenic HEK-Blue LPS detection sytem (Invivogen, UK) and confirmed that the LPS contamination was <0.05EU/ml.

A micellar formulation of a synthetic MPLA analogue has been denoted previously as IDRI-AQ001 and is more generally denoted as GLA-AF [18]. The biological and physicochemical characterization of GLA-AF has been published previously [7]. Resiquimod (R848) (Invivogen, UK) was reconstituted in endotoxin free water and one or both of these adjuvants were admixed with the recombinant proteins prior to administration.

### Minipigs, Immunization and Sampling

Ellegaard Göttingen minipigs were used in all experiments (Dalmose, Denmark). These pigs are a genetically managed strain of animals originally bred in the 1960s by combining Vietnamese potbelly, Minnesota Minipig and German Landrace lines, and maintained as a stable genetically coherent but outbred population. We first immunized five groups of female minipigs (n = 4 per group) aged 20 weeks with protein antigens and a dose titration of the TLR7/8 agonist adjuvant R848 or the aqueous formulation TLR4 agonist adjuvant GLA-AF (Table 1). Each adjuvant was tested for potency after topical mucosal application to the IN membrane and parenterally with an ID injection. We then combined a vaccine antigen with the optimal quantity of R848 (50 μg) together with increasing doses of GLA-AF to determine the optimally effective adjuvant combination (Table 2). The relative contribution of the addition of GLA-AF adjuvant to an optimal R848 dose was also assessed after an IN (mucosal) or ID (systemic) inoculation. Each antigen was administered twice in the presence of the adjuvant formulations with an interval of four weeks. Inoculations were staggered and alternate routes used each week to allow for completion of the phase 1 vaccination schedule in 8 weeks while preventing cross-stimulation with residual adjuvants. Likewise, the phase 2 schedule was completed after a further 2 months. The studies utilizing the model antigen CN54 HIVgp140 used seven animals per adjuvanted group with 3 control animals that received the antigen without adjuvantation. The pigs were vaccinated at three week intervals to allow development of somatically hypermutated antigen-reactive B cells in the draining lymph nodes. Each antigen dose was administered in a total volume of 200 μl, 100 μl into each nostril or 100 μl in two sites into the dermis of the pig ear. Pigs were sampled weekly. Blood drawn from the carotid artery was allowed to clot at room temperature for 30 min then centrifuged at 500 × g for 10 min and the serum removed and stored in aliquots at -80°C. Vaginal washes were performed by inserting a 1 cm bore tube into the vagina and washing the vault with 3 ml of 2 × PBS (FBB), then aspirating the wash fluid and adding 1/10 recovered volume of 10 × protease inhibitor cocktail set I (Millipore, UK). The vaginal wash sample was then centrifuged through a Spin-X column to remove large debris and frozen at -80°C. Nasal samples were taken using a Wek-Cel sponge (Beaver-Visitec, UK) from the mucosal membrane of the pig nares. The sponge sample was placed into a Spin-X column and the antibody eluted from the sponge using a high salt elution, effectively a 2 × PBS solution containing protease inhibitors. The sample was then centrifuged through a Spin-X column to remove large debris and frozen at -80°C.

**Table 1:** Co-formulated antigens and single adjuvant combinations. Each antigen was injected by the route indicated in groups of Göttingen minipigs (n = 4). The dose of adjuvant co-formulated with each antigen is detailed as a μg quantity given in a final volume of 100 μl.

Route	Antigen	Adjuvant	Group 1	Group 2	Group 3	Group 4	Group 5
IN	KLH (50ug)	R848	0 μg	50 μg	100 μg	200 μg	400 μg
ID	HIV Nef (20ug)	R848	0 μg	25 μg	50 μg	100 μg	200 μg
IN	TT (50ug)	GLA	0 μg	5 μg	10 μg	20 μg	40 μg
ID	OVA (20ug)	GLA	0 μg	2.5 μg	5 μg	10 μg	20 μg

**Table 2:** Comparison of combined R848 and titrated GLA-AF adjuvants. A constant quantity of the R848 adjuvant was administered via either the IN or ID routes together with increasing quantities of the GLA-AF adjuvant in final administration volumes of 100 μl.

Route	Antigen	Adjuvant	Group 1	Group 2	Group 3	Group 4	Group 5
ID	ESAT6 (20 μg)	R848 (50 μg) + GLA	GLA 0 μg	GLA 2.5 μg	GLA 5 μg	GLA 10 μg	GLA 20 μg
IN	B-gal (50 μg)	R848 (50 μg) + GLA	GLA 0 μg	GLA 5 μg	GLA 10 μg	GLA 20 μg	GLA 40 μg

### Antigen-Specific Antibody Semi-Quantitative ELISA

Serum and mucosal antigen-specific binding antibodies against the various recombinant proteins were measured using standardized ELISAs. Maxisorp high binding 96-well plates were coated with 100 μl recombinant proteins at 5 μg/ml in PBS overnight at 4°C. The standard immunoglobulins were captured with either a goat anti-pig IgG Fc (plate coated with 100 μl of a 1:2400 dilution) or a goat anti-pig IgA (100 μl of a 1:2000 dilution) (Bethyl laboratories, UK). These capture antibodies were coated onto the Maxisorp plates overnight at 4°C. Coated plates were washed three times in PBS-T before blocking with 200 μl PBS-T containing 1% bovine serum albumin for 1 hour at 37°C. After further washing, sera diluted 1/100, 1/1000 and 1/10,000 or mucosal wash samples diluted 1/10, 1/50 and 1/250 were added to the antigen coated wells and a standard titration of pig IgG or IgA immunoglobulin standards added to the respective capture antibody coated wells at 50 μl/well and incubated for 1 hour at 37°C. Plates were washed four times before the addition of 100 μl of a 1/6,000 dilution of goat anti-pig IgG-HRP or a 1/8,000 dilution of goat anti-IgA biotinylated secondary antibody and incubated for 1 hour at 37°C. The plates were washed four times and developed with 50 μl/well of KPL SureBlue TMB substrate (Insight Biotechnology, UK). The IgA isotype, that was biotin labeled, required a further streptavidin-HRP (R&D systems) amplification step prior to TMB development. The reaction was stopped after 5 min by adding 50 μl/well 1 M H_2_SO_4_, and the absorbance read at 450 nm on a VersaMax spectrophotometer using SoftmaxPro software.

### Antigen-Specific Avidity ELISA

The avidity indices of serum samples were determined by their antibody-antigen binding resistance to 8 M urea. Serum samples were pre-diluted to give an OD_450 nm_ readout between 1.0 and 1.5 in an ELISA and were added to CN54gp140 (5 μg) coated plates. The wells were then washed three times with either PBS-T or 8 M urea in PBS-T, before incubating with an anti-pig IgG-HRP secondary antibody. The plates were developed with TMB as described above. The avidity index was calculated as the percentage of average urea treated OD_450 nm_/average PBS-T OD_450 nm_. Antisera with index values exceeding 50% (retention in binding antibody) were ascribed to have high avidity, 30–50% were ascribed as intermediate avidity and <30% were considered to have low avidity binding.

### Virus Neutralisation Assay

HIV-1 serum neutralization assays were performed using a luciferase-based assay in TZM.bl cells as described previously [19]. Briefly, 3-fold serial dilutions of serum samples in 10% D-MEM growth media (100 μl/well) were performed in duplicate in 96 well plates. A 50 μl volume of virus was added to each well and the plates incubated for 1 hour at 37°C. TZM.bl cells in growth media (1x10^4^ cells/well per 100 μl volume) containing 11 μg/ml DEAE-Dextran (Sigma, USA) were then added to each well. The neutralization assay used replicate wells of TZM.bl cells alone (cell control) and TZM.bl cells with virus (virus control). Following a 72 hour incubation at 37°C, supernatant was removed, and cells were washed with sterile PBS, then treated with 1 × Reporter Lysis buffer (Promega) and frozen at -80°C for a minimum of two hours to lyse cells. A 50 μl volume of lysate was mixed with 50 μl of Luciferase Assay system substrate and luminescence was measured using Fluostar spectrophotometer, using Omega software (BMG Labtechnologies). The 50% inhibitory concentration (IC50) titer was calculated as the serum dilution that caused a 50% reduction in relative luminescence units (RLU) compared to virus control wells after subtraction of cell control RLUs. The viral strains used in the neutralization assays were CN54 (homologous) and ZM197 Clade C viruses, the same viral clade as the vaccine antigen.

### Statistical Analysis

The mean serum, mucosal antibody and avidity indices were compared using two-tailed Mann Whitney non-parametric tests or an unpaired t-test with Welch’s correction if the data variance was not equal.

## Results

### Molecular Adjuvants R848 and GLA-AF Promote Different Immune Outcomes when Administered by ID or IN Routes

We first assessed the capacity of R848 or GLA to enhance antigen-specific humoral immune responses in the minipig model on co-administration with a range of purified protein antigens. Different antigens were used to allow the overlapping study of the different routes of delivery whilst reducing animal usage, the antigens selected are common model antigens.

Adjuvants were titrated by route of administration to establish an effective dose for each molecular adjuvant, and animals were vaccinated at 0 and 4 weeks (Table 1). Antibody responses to each model antigen were measured by semi-quantitative antigen-specific ELISA and compared to control animals that received antigen in the absence of any adjuvant. The lowest dose of R848 (50 μg) administered by either the IN and ID routes significantly augmented the antigen-specific IgG immune response one week after boost vaccination (week 5, *p* = 0.0286) with no further advantage seen at higher doses (S1 and S2 Figs). Subsequently, a 50 μg dose of R848 was selected for all further investigations. Antigen-specific IgA responses were only observed following IN administration and were 1–2 logs lower than those seen for IgG. GLA-AF enhanced serum specific IgG responses when administered ID, with maximal effects in the 10–20 μg range. However, GLA-AF provided no apparent adjuvantation when administered IN and induced no detectable specific IgA responses following ID or IN administration. (S3 and S4 Figs).

**Fig 1:**
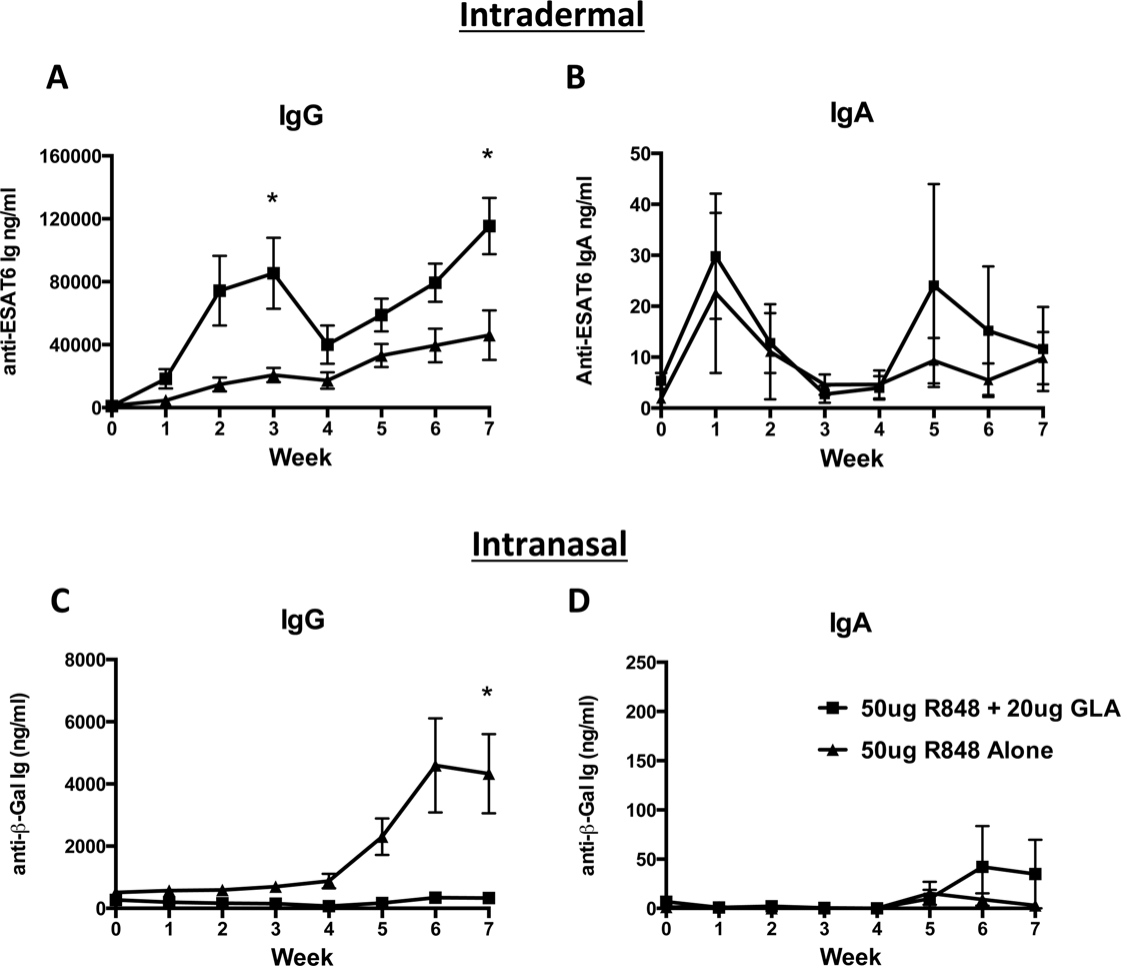
GLA-AF enhances R848 induced humoral responses after ID injection. A) The TLR4 agonist GLA-AF (20 μg) increased the TLR7 (R848–50 μg) adjuvanted ESAT-6 (recombinant *M*. *tuberculosis* early secreted antigenic target-6 kDa) antigen-specific IgG responses four-fold after a single prime and one boost ID vaccination (* *p* = 0.0335; Week 3 and *p* = 0.0338; Week 7). B) The combination of GLA-AF and R848 had no effect on serum IgA responses over R848 alone. C) The TLR4 agonist GLA-AF significantly ablated the R848 induced β-Gal (Beta galactosidase) antigen-specific IgG responses after IN injection (* *p* = 0.0261; Week 7). D) The combination of GLA-AF and R848 as well as R848 alone poorly augmented antigen-specific β-Gal serum IgA responses.

Using a fixed dose of R848 adjuvant (50 μg), we next assessed the potential impact of combining R848 with increasing concentrations of GLA-AF, when administered via the ID or IN routes (Table 2 and S5 and S6 Figs). A dose of 20 μg GLA-AF had a significant additive effect on antigen-specific antibody responses when combined with R848 and injected ID, achieving a 4-fold increase in peak mean specific IgG response compared with R848 alone (85.4 vs 20.8 μg/ml of specific antibody respectively (*p* = 0.0335)), three weeks after the primary vaccination, and a 2.5-fold increase three weeks after a boost inoculation (R848 + GLA (115.4 μg) vs R848 alone (46.1 μg) (*p* = 0.0338)). It remains unclear when comparing the GLA-AF augmentation of the response to ID administered OVA antigen and the combination ESAT-6 as to whether R848 provided any benefit to the GLA-AF induced response. Further studies to clarify the relative contribution of GLA-AF and R848 will be needed. The TLR ligands either alone or in combination had minimal effect on circulating antigen-specific IgA when administered by ID injection (Fig 1A and 1B, S5 Fig). A contrasting picture was observed with IN administration, where a 20 μg dose of GLA-AF completely suppressed R848-induced antibody immune responses after IN inoculation (*p* = 0.0261; Week 7) (Fig 1C and 1D). Furthermore, addition of as little as 5 μg of GLA-AF was sufficient to reduce adjuvantation by R848 (S6 Fig; *p* = 0.0315–50 μg R848 alone vs 50 μg R848 + 5 μg GLA-AF; Week 7). Based on these initial findings we selected 50 μg R848 plus 20 μg GLA-AF as an optimal combination for ID vaccination and 50 μg R848 alone for IN inoculation.

### Mixed Route Prime-Boost Combinations Elicit High Levels of CN54gp140-Specific Humoral Immunity

Using the adjuvant doses selected above we next investigated the impact of mixed route prime-boost combinations on the induction of systemic and mucosal antibody responses using a model HIV envelope glycoprotein antigen, CN54gp140 [20]. Responses were compared to antigen administered in the absence of adjuvant that typically elicits only low-level systemic immunity [21–23]. We compared the immune outcomes in animals that received 3 × IN priming vaccinations followed by 2 × ID booster vaccinations (Group A) to animals that received 3 × ID priming vaccinations followed by 2 × IN boosts (Group B), each administered with three-week intervals between each vaccination (n = 7 per group).

**Fig 2:**
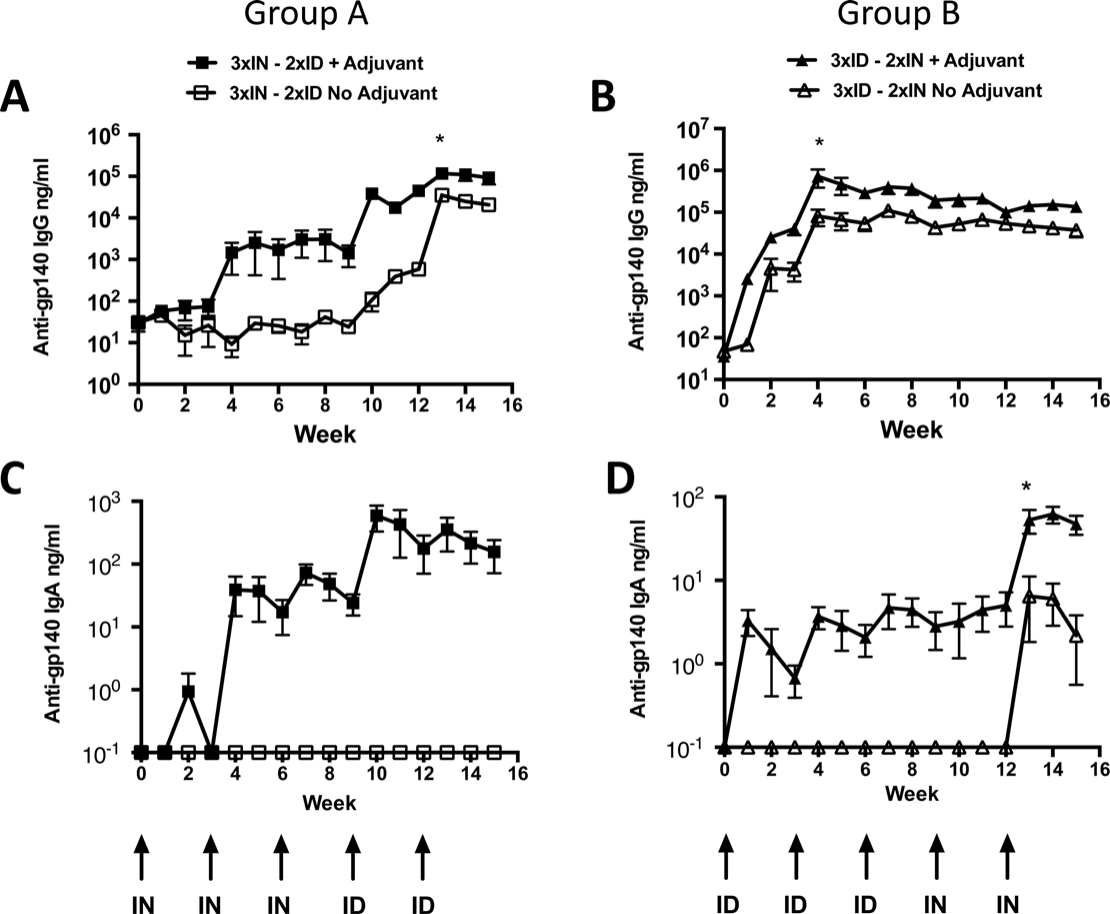
Combinations of R848 and/or GLA-AF administered by each route significantly enhance HIV Env gp140 antigen-specific serum immunoglobulin responses. A) Animals that received three priming inoculation via the IN route followed by two ID boost injections (Group A) exhibited significantly augmented serum IgG responses compared to animals that received the unadjuvanted vaccine (*p = 0.0167; Week 13). B) Animals that received three priming inoculations via the ID route followed by two IN boost injections (Group B) exhibited significantly augmented serum IgG responses compared to animals that received the unadjuvanted vaccine (*p = 0.0167; Week 4). C) Group A animals generated antigen-specific IgA in response to IN inoculation which was boosted by the first but not the second ID injection. Unadjuvanted IN administration failed to elicit detectable responses and no boosting or anamnestic response was observed upon ID vaccination. D) Group B animals demonstrated low level IgA generation in response to adjuvanted ID injections and this was boosted by subsequent IN inoculations (*p = 0.037; week 13).

Animals in group A, receiving three initial IN inoculations with CN54gp140 antigen combined with R848 (50 μg) elicited mean serum antigen-specific antibody levels of 3 μg/ml. These responses were significantly enhanced by subsequent ID injections, adjuvanted with the R848 (50 μg) + GLA-AF (20 μg) combination, rising to a peak value of 116 μg/ml CN54gp140-specific IgG (mean) one week after the second ID vaccination (Fig 2A). In contrast, animals that received IN protein in the absence of adjuvant failed to mount any detectable response to IN administration but displayed some evidence of an anamnestic response after the first ID injection, rising from a mean of 25 ng/ml antigen-specific Ab to 582 ng/ml, suggesting that the three IN inoculations in the absence of adjuvantation were seen by the immune system but had little immunological impact. However, one week following the second ID immunization, serum antibody levels (35 μg/ml) were approximately 1/3 that of the adjuvanted group but were significantly lower (*p* = 0.0167; Week 13) (Fig 2A). For IgA, animals that received vaccination in the presence of adjuvant demonstrated detectable levels of antibody. The level of vaccine antigen-specific IgA generated in the serum was low, reaching a peak of 590 ng/ml at week 10, one week after the first ID inoculation. The unadjuvanted animals had no specific IgA response (Fig 2C).

Conversely, animals in group B, receiving three initial ID injections co-administered with combined adjuvants (R848 (50 μg) + GLA-AF (20 μg)), displayed robust CN54gp140 antigen-specific IgG responses with mean serum IgG responses reaching 719 μg/ml, one week after the second ID administration with subsequent ID and IN vaccinations providing no additional augmentation of response. This was 6-fold higher than those following three IN and 2 × ID administrations in Group A (Fig 2A). Animals in Group B that received 3 × ID + 2 × IN immunizations with CN54 gp140 in the absence of adjuvantation demonstrated reasonable antigen-specific antibody elicitation after the second and third ID injection, with a mean peak serum antigen specific IgG concentrations of 109 μg/ml (Fig 2B), similar to the maximum peak achieved with the unadjuvanted Group A 3 × IN + 2 × ID schedule (Fig 2A). This 3 × ID + 2 × IN regimen also generated vaccine antigen-specific IgA responses, although ten-fold lower than the Group A regimen, reaching a peak of 53 ng/ml at week 13 and was significantly elevated over the unadjuvanted regimen (*p* = 0.0370)(Fig 2D).

When comparing the antibody profile of the two prime boost regimes (Group A vs B), although each group provided an initial advantage with respect to serum IgA or IgG induction (group A and B respectively), by the end of each series of vaccinations the immune outcome in both converged to similar serum antibody levels for both IgG and IgA (S7 Fig). This was also the case for the non-adjuvanted controls, although the absolute levels of elicited specific IgG levels were significantly reduced in comparison to those in the adjuvanted groups (IgG week 14 adjuvanted vs unadjuvanted *p* = 0.0167). IgA was mostly below the limits of detection in the unadjuvanted groups (S7 Fig).

**Fig 3:**
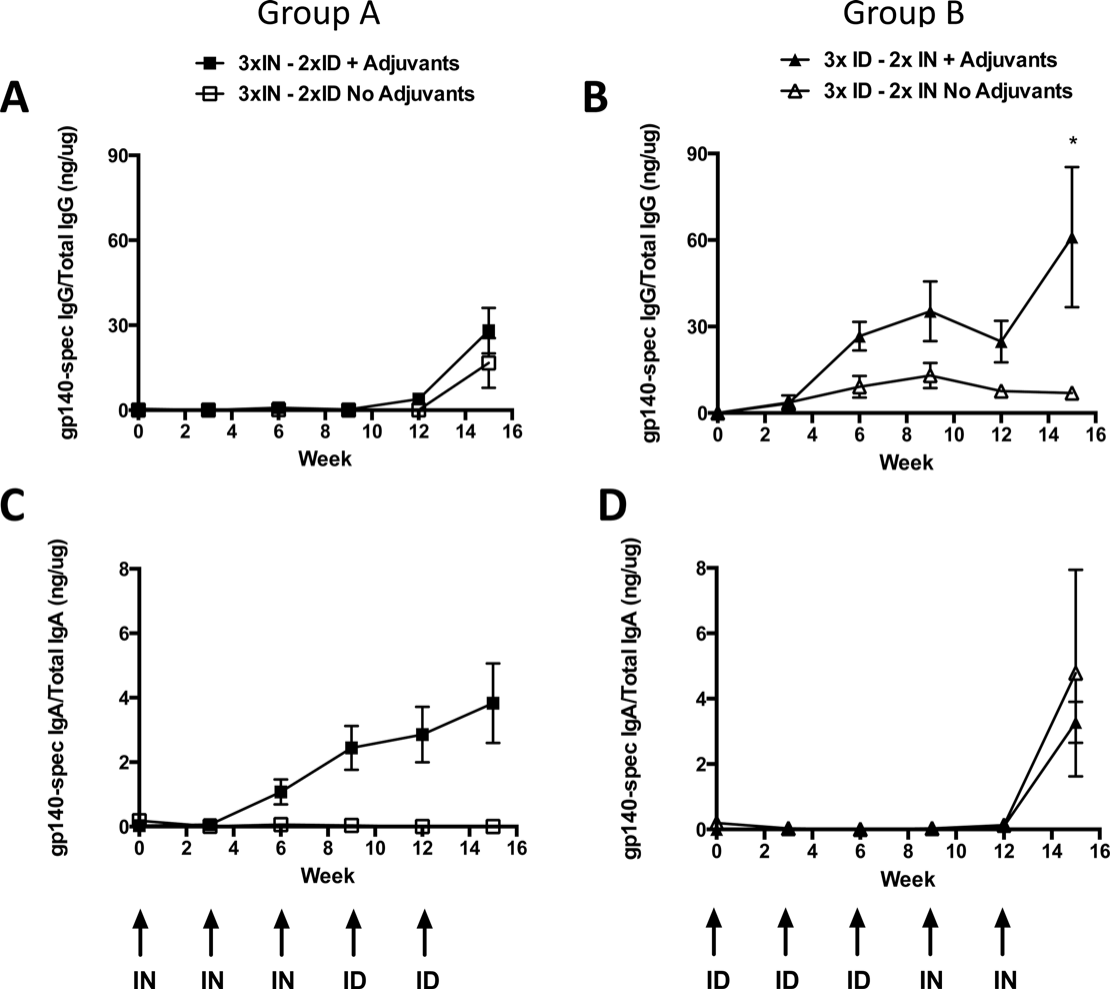
Combinations of R848 and/or GLA-AF administered by each route significantly enhance HIV Env gp140 antigen-specific nasal mucosal immunoglobulin responses. A) Group A (3 × IN + 2 × ID) animals had undetectable antigen-specific nasal IgG until after the first ID vaccination While slightly elevated these responses were essentially indistinguishable from animals that received the unadjuvanted vaccine. B) Group B (3 × ID + 2 × IN) animals demonstrated higher nasal mucosal IgG compared to animals that received the unadjuvanted vaccine (*p = 0.0167; Week 15). C) IN inoculation elicited antigen-specific IgA that was boosted intranasally after ID vaccinations. D) ID vaccinations did not elicit any detectable antigen-specific IgA in the nasal cavity. Subsequent IN inoculation generated low levels of specific IgA.

We then measured mucosal antibody present in the IN cavity, with samples being taken before inoculation to prevent complications that might arise from potential adjuvant induced mucosal inflammation as this was a site of administration (Fig 3A–3D). The measured levels of specific IgG were low but mirrored the elicitation profiles observed in serum leading us to conclude that mucosal antibody likely resulted from serum exudation into the nasal cavity rather than being locally produced. Animals in Group A failed to induce detectable local specific IgG responses following 3 × IN primes, however responses were induced on subsequent ID boosts. In contrast, animals in Group B that received 3 × ID priming vaccinations achieved statistically significant higher levels of specific IgG per μg of total IgG than unadjuvanted animals (Fig 3B; *p* = 0.0167; week 15) and also animals in Group A receiving 3xIN inoculations (S8 Fig; *p* = 0.0006). Furthermore, the second IN boost rather than the first in Group B enhanced ID primed antigen-specific IgG response (Fig 3B).

A different pattern was observed for local IgA responses: here, 3 × IN priming inoculations (Group A) were able to elicit local specific IgA that was maintained, though not enhanced by subsequent ID injections. Furthermore, although ID priming in Group B had no impact on apparent local specific IgA induction, 2 × IN boosts were sufficient to induce similar local levels of specific IgA to those in Group A (Fig 3C and 3D).

Thus mirroring serum responses, the final IN antigen-specific antibody responses in both groups were essentially the same, approximately 30 ng gp140-specific IgG per 1 μg of total IgG present, or 3% of the available IgG, irrespective of the order of administration. However in the mucosal nasal site the antigen-specific IgA was only 10-fold lower than IgG and constituted around 0.3% of the total IN IgA and not 1000-fold lower as observed in the serum, indicating a mucosal IgA bias of antigen-specific secreted IgA (Fig 3C and 3D).

**Fig 4:**
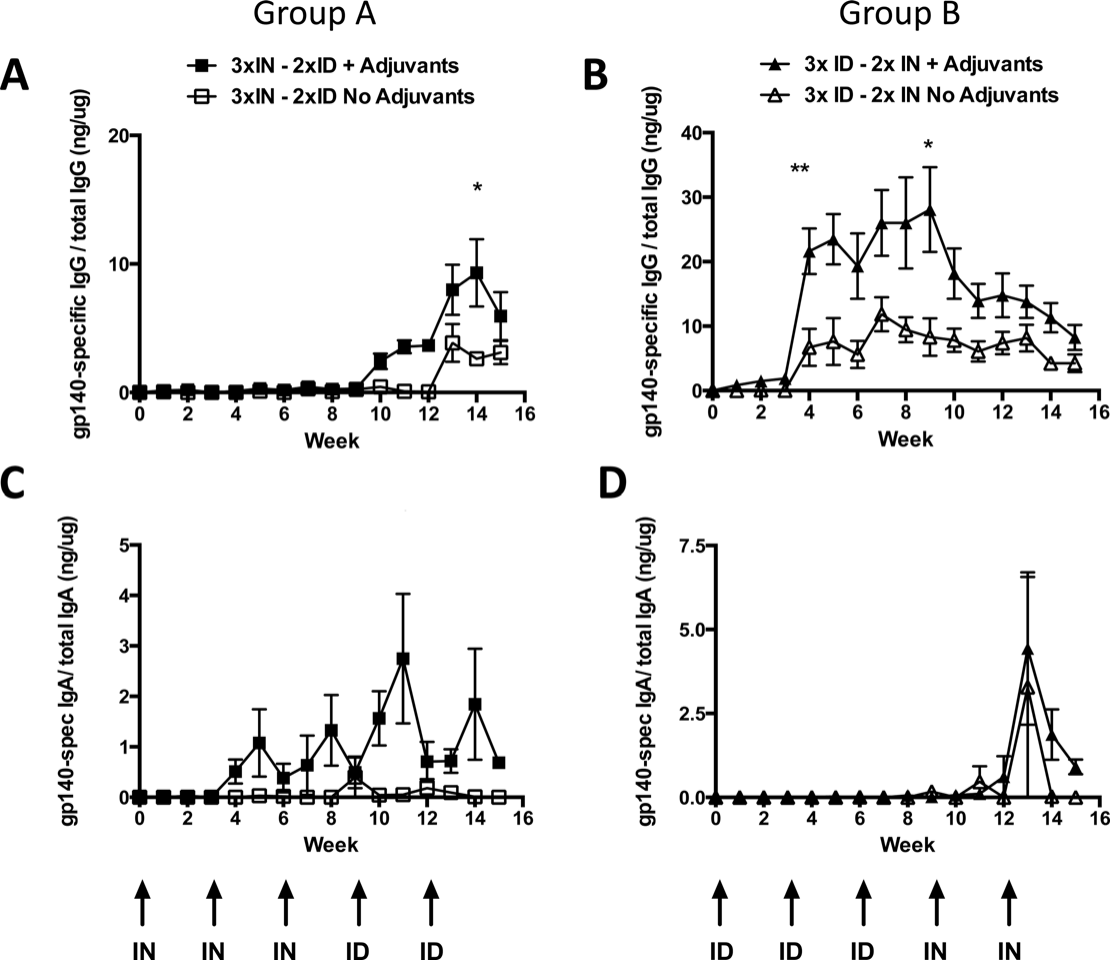
Combinations of R848 and/or GLA-AF administered by each route significantly enhance HIV Env gp140 antigen-specific vaginal mucosal immunoglobulin responses. A) Group A (3 × IN + 2 × ID) animals exhibited augmented vaginal mucosal IgG levels compared to animals that received the unadjuvanted vaccine (*p = 0.0167; week 14). B) Group B animals had higher vaginal mucosal IgG compared to animals that received the unadjuvanted vaccine (**p = 0.0069; week 4 and *p = 0.0247; week 9). C) IN inoculations elicited antigen-specific IgA that was boosted by a second and third IN application and by the first ID vaccination. D) ID vaccinations failed to elicit detectable vaginal IgA, subsequent IN inoculations generated very low levels of antigen-specific IgA.

As we were interested to explore the potential immunological linkage between the nasal associated lymphoid tissue (NALT) and the lower genital tract [24, 25], being the primary site of HIV infection in women, we also assessed antigen-specific antibody response in vaginal lavage. Levels of specific antibody detected were similar in quantity to that measured in the nasal cavity (Fig 4A and 4B). Animals in Group A receiving 3 × IN + 2 × ID only elicited antigen-specific vaginal IgG responses after ID boosts, where responses were higher in the adjuvanted group (*p* = 0.0167; Week 14). Animals in Group B that initially received three adjuvanted ID immunisations exhibited the highest levels of specific antibody per μg of total IgG present in the vaginal wash (Fig 4B; *p* = 0.0069; week 4 and *p* = 0.0247; week 9), however, these responses rapidly waned and were not maintained or enhanced by subsequent IN vaccine boosts. This contrasts with the nasal cavity where the second IN boost was able to increase the local IgG (Fig 3B). Vaginal IgA was boosted by the second and third IN inoculation as well as by the first ID injection, reaching a peak level of 3 ng/μg total IgA. The second ID injection had no further boosting effect (Fig 4C). In contrast, the ID injections in Group B had little ability to generate vaginal IgA responses with antigen-specific IgA only being detectable after the second IN inoculation (Fig 4B). Again, both route regimens achieved similar mucosal antigen-specific immunoglobulin profiles at the end of each vaccination schedule. IgG reached a level of 10 ng specific IgG antibody per 1 μg of total IgG antibody indicating that approximately 1% of the IgG antibody present in the vaginal vault was specific for the vaccine antigen, and approximately 1 ng of antigen-specific IgA per 1 μg total or 0.1% of the IgA. Thus, antigen-specific IgA mucosal levels were again 10-fold lower than mucosal IgG, in contrast to the larger differential in the serum compartment, and were elicited in response to IN prime (Group A) or boost (Group B) immunizations (Fig 4C and 4D).

**Fig 5:**
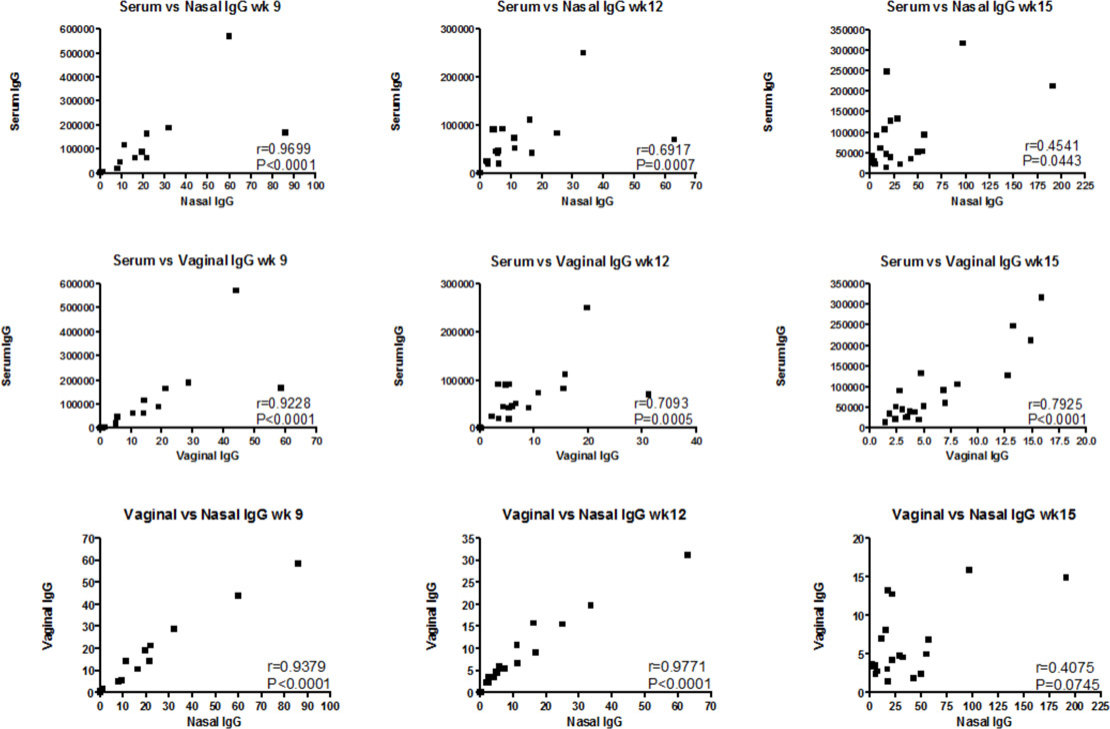
Spearman Rank correlations comparing serum, nasal and vaginal mucosal IgG. A strong positive correlation was found at week 9 and 12 between the amounts of antigen-specific antibody present at each site but this relationship was lost by the end of the vaccination regimen as the nasal compartment exhibited increased levels of specific IgG while vaccine-specific antibody decreased in the serum and vaginal vault. By week 15, 3 weeks after the final vaccinations, the IgG present in the serum and vaginal samples still correlate strongly while that in the nasal compartment exhibits little or no significant relationship.

A Spearman rank correlation was carried out to test the relationship between the vaccine antigen-specific antibody levels measured in each of these compartments, the vaginal vault, the IN cavity and the serum. A strong correlation was observed between all the compartments at week 9 (after the initial 3xIN or 3xID vaccinations) and after week 12 (post the first boost by the alternate route). However, at the end of each schedule the IgG levels in the nasal compartment began to separate from the serum and vaginal sites, losing the strong degree of correlation with the serum and failing to correlate with the IgG in the vaginal compartment. The second and final IN administration increased the specific IgG antibody present in the nasal cavity while the vaginal and serum levels continued to decrease, perhaps suggesting a local increase of antibody producing cells at the site of vaccination (Fig 5).

### Qualitative Assessment of Antigen-Specific Antibodies Elicited by the Alternate Route Regimens

**Fig 6:**
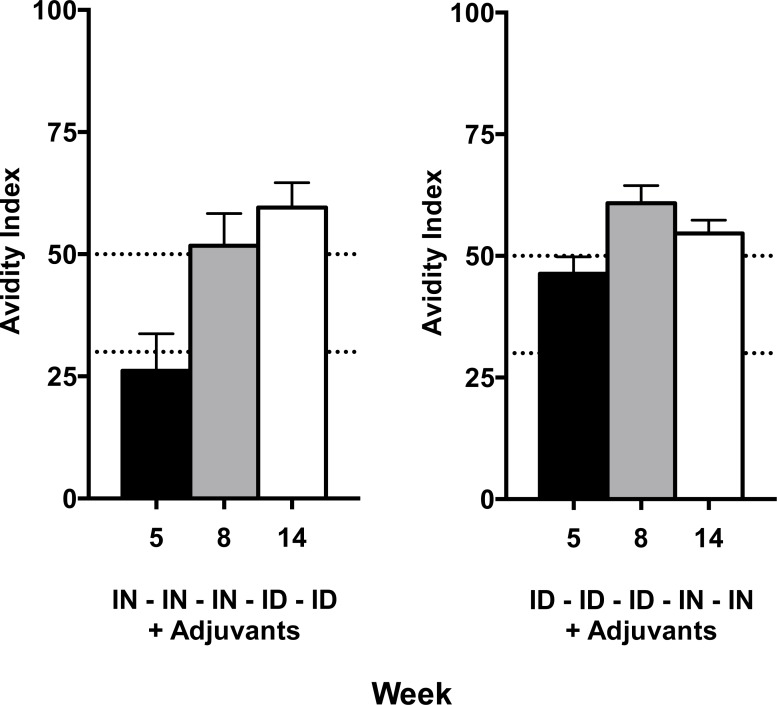
The antigen-specific polyclonal sera from the different regimens exhibited differing avidity indices throughout the course of the vaccinations. Low avidity = <25%, medium avidity = 25–50% and high avidity = >50%. After two priming vaccinations (week 35) the sera from animals inoculated via the IN route had low avidity binding to the vaccine antigen while animals that received ID injections had medium–high avidity gp140-antigen reactive sera. Optimally adjuvanted ID injections were able to continue to enhance avidity while the IN inoculation failed to enhance or even maintain the previous high level avidity elicited by prior ID injections.

**Fig 7:**
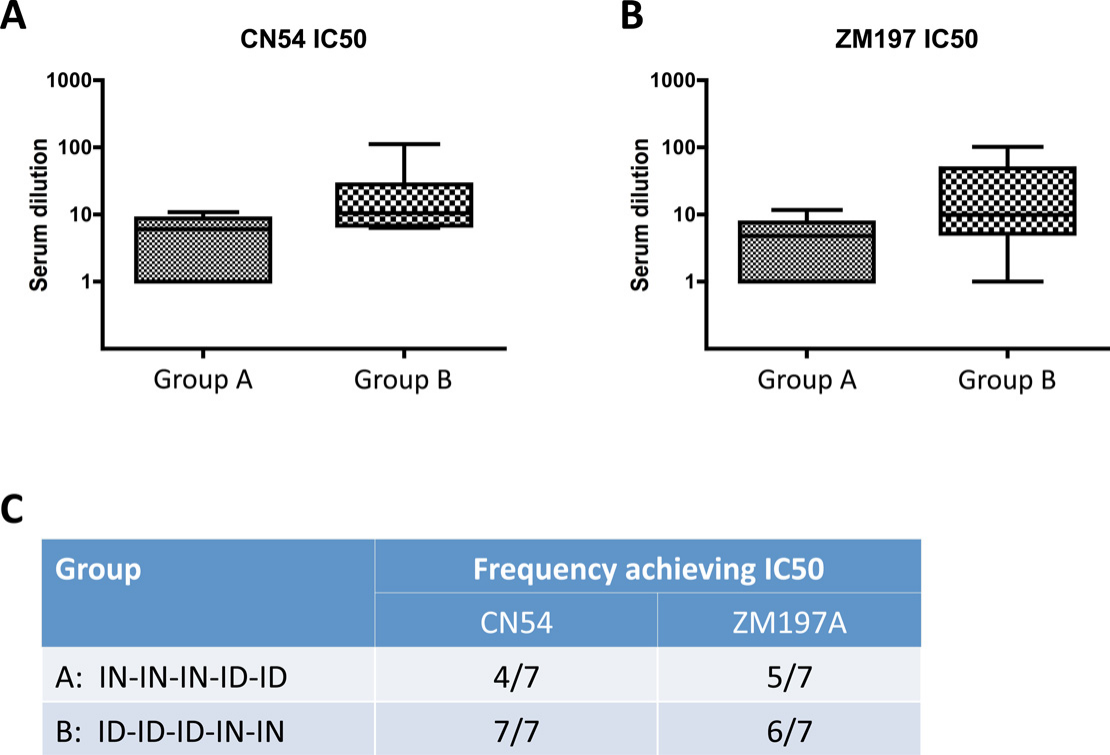
Sera from each group of animals were tested in a functional HIV neutralising TZM-bl assay. The serum dilution that was able to achieve half maximal inhibition of HIV infectivity was higher in Group B (3 × ID + 2 × IN) for both a closely sequence matched Clade C virus CN54 (A) and a more sequence divergent Clade C HIV (B). All animals from this group achieved neutralisation compared to only 4 of the 7 animals in Group A (3 × IN + 2 × ID) (C).

As each regimen elicited similar quantitative levels of CN54gp140 antigen-specific serum and mucosal antibody at the end of the vaccine regimen we next assessed whether the serum antibodies generated from each schedule had the same or different qualitative characteristics. Although functional assays for polyclonal pig anti-sera are limited we were able to assess the avidity and the neutralisation capacity of sera from each animal. Firstly, using a urea-denaturation avidity assay we determined that by week 5, after just two priming immunisations, the adjuvanted ID injections in Group B were able to elicit intermediate avidity antigen-specific responses, while in Group A serum antibodies elicited by two IN inoculations exhibited an overall low avidity index (Fig 6). Importantly, these assays measure the avidity index from an input-normalised quantity of specific antibody, as each individual sera is diluted to provide an ELISA OD reading of between 1 and 1.5, the linear part of the titration curve. At week 8, after the first three inoculations via the initial route and at week 14, at the end of the respective schedules, we noted that both regimens elicited sera with a high avidity index with no statistical difference between the groups. The animals in Group A that were primed intranasally and boosted by ID injections continued to show a slight upward trend towards higher avidity antisera, while animals in Group B that were primed intradermally and boosted by IN inoculations demonstrated a slight decrease in the overall sera avidity suggesting that the ID route was able to maintain high avidity serum indices generated after vaccination while the IN route was less able to do so (Fig 6). We also determined whether the sera samples from each animal exhibited any differential capacity to neutralise two HIV-1 strains in a TZM-bl viral infectivity assay. We used CN54 (homologous) and ZM197 Clade C viruses which are the same viral clade as the vaccine antigen. Sera from both animal groups exhibited some capacity to prevent viral infection with every animal in Group B (3 × ID + 2 × IN) being able to reduce viral infectivity by 50%, while in Group A, 4 of the seven animals displayed the same level of measureable inhibition against the CN54 homologous clade C virus. Overall, the viral inhibition capacity from the sera of each group of animals was not statistically distinguishable (Fig 7).

## Discussion

In this study we explored the potential utility of R848 and GLA-AF adjuvants and their combination for augmentation of humoral immune responses after inoculation via the ID and/or IN routes.

Although TLR adjuvant combinations may reveal unique properties of immune potency or efficacy, these can sometimes be exhibited differently in rodents when compared to nonhuman primates (NHP) or humans [26]. While the major DC subsets have been described in both mouse and man there are considerable differences between these species’ DCs, particularly in relation to TLR expression and critically the lack of a functional TLR8 gene in mice [27–29]. The differential potential of TLR4 and TLR7/8 agonists for mucosal and parenteral adjuvantation has not been previously established in an appropriate animal model. On an anatomic level there is considerable interest in the exploitation of the potential common mucosal immune system linkage between the NALT and lower female genital track, a primary portal of entry for HIV and other sexually transmitted infections. This phenomenon has been clearly and reproducibly demonstrated in mice, but there is limited evidence in larger animals including humans [24, 30]. To investigate this common mucosal linkage we compared the IN route, known to generate vaginal immune responses in mice [25, 31], to the ID route which typically does not elicit appreciable vaginal mucosal antibody responses. Knowing that different vaccination routes for priming and boosting can modulate magnitude, quality, and localization of the immune response, at least in mice [32], we further assessed the impact of mixed route (IN & ID) prime-boost combinations and their order on elicited mucosal responses.

Ethical considerations and expense make it impractical to screen all potential adjuvants and route combinations in non-human primates; therefore, we looked to utilise an intermediate animal model that might be more predictive of human responses than rodent species. We chose to use the Göttingen minipig as an animal model on the basis of closer similarity with respect to functional expression of TLR molecules (and in particular TLR4 and 8) [15–17, 33–35], immune system responses, and skin anatomy to humans than that of rodent species [36–38]. Furthermore, the NALT of pigs, unlike rodents, has many similarities to that found in humans, displaying multiple lymphoid nodules in the nasopharygeal region, specifically the pharyngeal tonsils, analogous to the Waldeyer's ring in humans [39, 40]. In addition, the larger size of minipigs is advantageous for continuous sampling of serum and mucosal immune responses from individual animals. The early phase of the study adopted a novel matrix design to facilitate screening of a larger number of adjuvant concentrations administered via the ID and IN routes, thereby reducing animal usage and refining the quality of data produced.

TLR7/8 (R848) and TLR4 (GLA-AF) agonist adjuvant combinations augmented antigen-specific antibody immune responses after an ID vaccination but conversely GLA-AF dampened R848-induced response after IN administration. These discrepant results could simply reflect a physical impact of GLA-AF on IN absorption of R848. However, co-administration of GLA-AF did not inhibit R848 responses after ID vaccination, suggesting that the inhibitory effect of GLA-AF on R848-driven IN responses is likely an active process rather than a simple formulation issue. The inhibitory effects of GLA-AF when administered IN are in marked contrast to the strong adjuvant effects seen in mice when administered by the same route, furthermore R848 was a less potent adjuvant than GLA-AF when administered parentally [25]. These differences further highlight the potential limitations of the murine model for adjuvant selection.

Having established conditions for ID and IN administrations we then focused on the use of the CN54gp140 envelope protein from HIV-1 as a model antigen. In a multi-route vaccination regimen we found that ID vaccination effectively augmented serum IgG antigen-specific responses while IN inoculation was better at eliciting serum IgA, a bias that has been described in a number of studies [41, 42]. However, after three priming inoculations via one route followed by two boosts by the alternate route we noted that the quantities of serum antigen-specific antibody converged, and that the order of administration route did not have a significant effect on the overall antigen-specific serum Ig levels. The early lead in the generation of specific IgG observed in the ID vaccine group was not maintained, indeed the third ID priming vaccination failed to substantially enhance the level of serum antigen-specific immunoglobulin. However, a boosting effect was evident for IgG upon ID administration following IN priming and conversely for IgA upon IN boost inoculation following ID priming, reflecting the propensity of ID to enhance serum IgG and IN to elevate serum IgA. By utilizing these particular TLRs and administering via the IN and ID routes we had hoped to enhance antigen-specific mucosal immune responses by promoting lymphocyte (B and T cell) homing to the mucosae and mucosal associated lymphoid tissue. However, the antigen-specific antibody measured in vaginal lavage samples very closely mirrored the amount of specific antibody in the sera. These data suggest vaginal antibody levels are most likely reflective of serum exudation rather than localised response of resident or circulating mucosal-homing antigen-specific B cells. Indeed, we found no evidence of a common mucosal linkage between the vaginal and the nasal mucosae. After three systemic ID vaccinations we noted a strong correlation between the amount of specific antibody in the nasal and vaginal compartments based on serum transudation but this correlation was not maintained after IN boosting of the ID primed response, which boosted nasal vaccine-specific IgG levels but did not elevate specific IgG at the distal vaginal site. The Group B (3 × ID + 2 × IN) regimen exhibited increased specific IgG levels at the local nasal mucosal site after the second IN inoculation, while no increase was evident systemically, likely because the levels were already high. If there were a direct mucosal linkage, due to B cell mucosal homing between the nasal and vaginal mucosae, one could expect a concurrent increase in the vaccine-antigen specific IgG within the vaginal vault but this was not observed in these minipig studies, though a nasal-vaginal mucosal linkage is readily demonstrated in mice [21, 43, 44].

As each regimen elicited similar quantities of antigen-specific antibody with no observable impact from the order of administration (IM/IN vs IN/IM), we next assessed the quality of the antibodies by measuring the overall avidity of the antigen-reactive polyclonal sera. Although IN priming was slower to induce affinity maturation when compared to ID vaccination, by the end of the initial 3xIN or 3xID priming vaccinations the avidity indices achieved by either route were similar. We also assessed HIV neutralization as a functional outcome of vaccination regimens using two Clade C HIV viruses, one closely matching to the vaccine antigen (CN54). Animals from both the 3 × IN + 2 × ID and the alternate 3 × ID + 2 × IN groups exhibited similar levels of viral inhibition although there was a trend toward more animals reaching IC50 neutralisation levels in the 3 × ID + 2 × ID regimen. We did not test the nasal and vaginal samples for neutralising capacity due to low levels of antigen-specific antibody. We concluded that the antibodies generated from either regimen were essentially similar in quantity and quality.

These data begin to address important issues relating to adjuvant combinations and the route of administration. The observation that TLR4 can both enhance or suppress the adjuvant effects of TLR 7/8 according to the route of administration (IN versus IM) raises interesting questions. It is likely that these results reflect differences in innate sensing populations and TLR expression patterns between these two compartments. Whether these findings translate to NHP and humans warrants further study. Understanding how this difference modulates resultant adaptive immune responses may provide insight into the generation of systemic and mucosal responses to invading pathogens. Furthermore, these data serve to highlight the need to determine the potential impact of adjuvant combinations according to route of administration.

## Supporting Information

S1 Fig Specific antibody responses to R848 adjuvanted antigen administered IN. The TLR 7/8 adjuvant R848 administered via the IN route significantly augments antigen-specific serum IgG and IgA responses to the KLH (Keyhole Limpet Haemocyanin) vaccine antigen. A) Amounts of R848 ranging from 400 μg down to 50 μg were formulated with 50 μg KLH and administered directly into the pig nares, significance is shown at one week after a boost IN inoculation (*p = 0.0286; 50, 100 and 200 μg IgG and *p = 0.0353; 50 μg). B) The antigen-specific serum IgG and IgA responses of all adjuvanted groups compared to the unadjuvanted control.
(TIF)

S2 Fig Specific antibody responses to R848 adjuvanted antigen administered ID. The TLR 7/8 adjuvant R848 administered via the ID route significantly augments antigen-specific serum IgG responses to the Nef HIV viral antigen. A) Amounts of R848 ranging from 200 μg down to 25 μg were formulated with 50 μg Nef and injected ID into the skin at the back of the ear. These pigs exhibited very high anti-Nef IgA antibody background, probably due to cross-reactivity to an endogenous porcine retrovirus. B) Shows the Nef antigen-specific serum IgG responses of all adjuvanted groups compared to the unadjuvanted control.
(TIF)

S3 Fig Specific antibody responses to GLA-AF adjuvanted antigen administered IN. The TLR 4 adjuvant GLA-AF administered via the IN route weakly augments antigen-specific serum IgG but does not enhance IgA responses to the TT (Tetanus Toxoid Fragment c) vaccine antigen. A) Amounts of GLA-AF ranging from 40 μg down to 5 μg were formulated with 50 μg TT and administered directly into the pig nares. B) The antigen-specific serum IgG and IgA responses of all adjuvanted groups compared to the unadjuvanted control.
(TIF)

S4 Fig Specific antibody responses to GLA-AF adjuvanted antigen administered ID. The TLR 4 adjuvant GLA-AF administered via the ID route strongly augments antigen-specific serum IgG but does not enhance IgA responses to the OVA (Ovalbumin) vaccine antigen. A) Amounts of GLA-AF ranging from 20 μg down to 2.5 μg were formulated with 50 μg OVA and injected ID into the skin at the back of the ear, significance is shown at one week after a boost IN inoculation, significance is shown at one week after a boost IN inoculation (*p = 0.0286; 10 and 20 μg). B) Shows the antigen-specific serum IgG and IgA responses of all adjuvanted groups compared to the unadjuvanted control.
(TIF)

S5 Fig Adjuvant combinations administered ID. A fixed amount of TLR 7/8 R848 adjuvant was titrated against increasing quantities of co-formulated TLR 4 agonist GLA-AF and administered ID to the skin on the back of the ear. The combination of 50 μg R848 and 20 μg GLA-AF demonstrated significant augmentation of the ESAT-6 (Early secreted antigen target-6 from Mycobacterium tuberculosis) antigen-specific IgG response over that of ESAT-6 delivered with only R848 at both 3 weeks post the primary injection and three weeks after a boost vaccination (*p = 0.033).
(TIF)

S6 Fig Adjuvant combination administered IN. A fixed amount of TLR 7/8 R848 adjuvant was titrated against increasing quantities of co-formulated TLR 4 agonist GLA-AF and administered IN directly into the pig nares. The combination of 50 μg R848 and any quantity of GLA-AF completely ablated the R848 generated response to β-Gal.
(TIF)

S7 Fig A comparison of serum IgG levels elicited by each regimen. A) Adjuvanted ID injections provide an early enhancement over the IN route (*** *p* = 0.0006; Week 4) which was lost by the end of the regimen. B) Serum IgA levels are statistically increased in the IN vaccinated pigs (***p = 0.0006; Week 10) over the ID primed animals but this difference was lost at the end of the vaccination schedule.
(TIF)

S8 Fig A comparison of nasal mucosal immunoglobulin levels elicited by each regimen. A) Adjuvanted ID injections provided an early and statistically significant enhancement over the IN route (***p = 0.0006; Weeks 6, 9 and 12) which was lost by the end of the regimen. B) Nasal mucosal IgA levels are statistically increased in the IN vaccinated pigs (***p = 0.0006; Weeks 6, 9 and 12) over the ID primed animals but this difference was also lost at the end of the vaccination schedule.
(TIF)

S9 Fig A comparison of vaginal mucosal immunoglobulin levels elicited by each regimen. A) Adjuvanted ID injections provided an early and statistically significant enhancement over the IN route (***p = 0.0003; Weeks 5, 8 and 13) which was lost by the end of the regimen. B) Vaginal mucosal IgA levels are statistically increased in the IN vaccinated pigs (*p = 0.0256; Week 5, *p* = 0.0210; Week 11) over the ID primed animals but this difference was lost at the end of the vaccination schedule.
(TIF)
