# Supplementary figures and images for: TLR4 and TLR7/8 Adjuvant Combinations Generate Different Vaccine Antigen-Specific Immune Outcomes in Minipigs when Administered via the ID or IN Routes
Source: PLoS One. 2016 Feb 10;11(2):e0148984. doi: 10.1371/journal.pone.0148984 (PMC4749393; doi:10.1371/journal.pone.0148984)

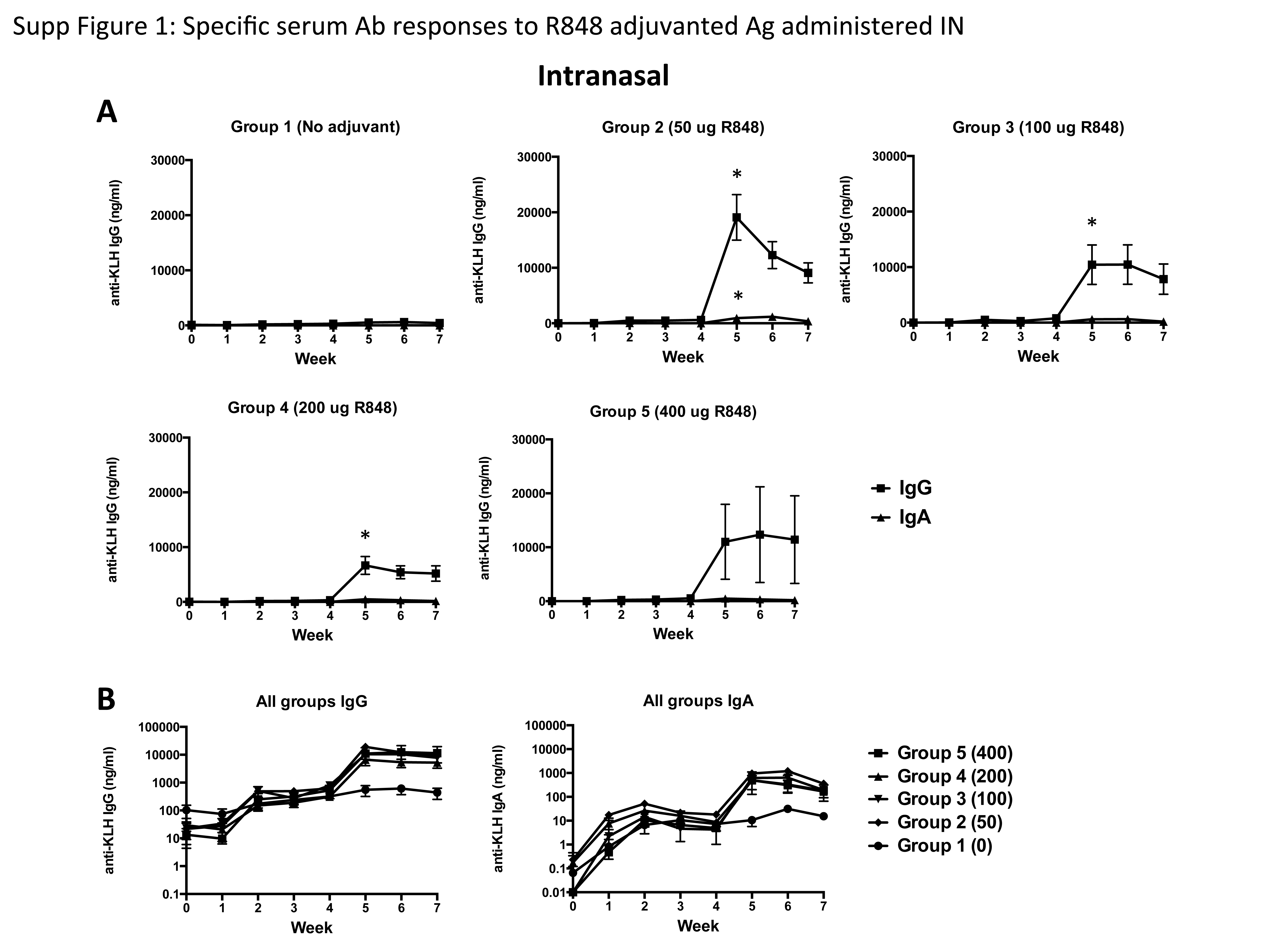

Supplement: S1 Fig — The TLR 7/8 adjuvant R848 administered via the IN route significantly augments antigen-specific serum IgG and IgA responses to the KLH (Keyhole Limpet Haemocyanin) vaccine antigen. A) Amounts of R848 ranging from 400 μg down to 50 μg were formulated with 50 μg KLH and administered directly into the pig nares, significance is shown at one week after a boost IN inoculation (*p = 0.0286; 50, 100 and 200 μg IgG and *p = 0.0353; 50 μg). B) The antigen-specific serum IgG and IgA responses of all adjuvanted groups compared to the unadjuvanted control. (TIF) [file pone.0148984.s001.tif]

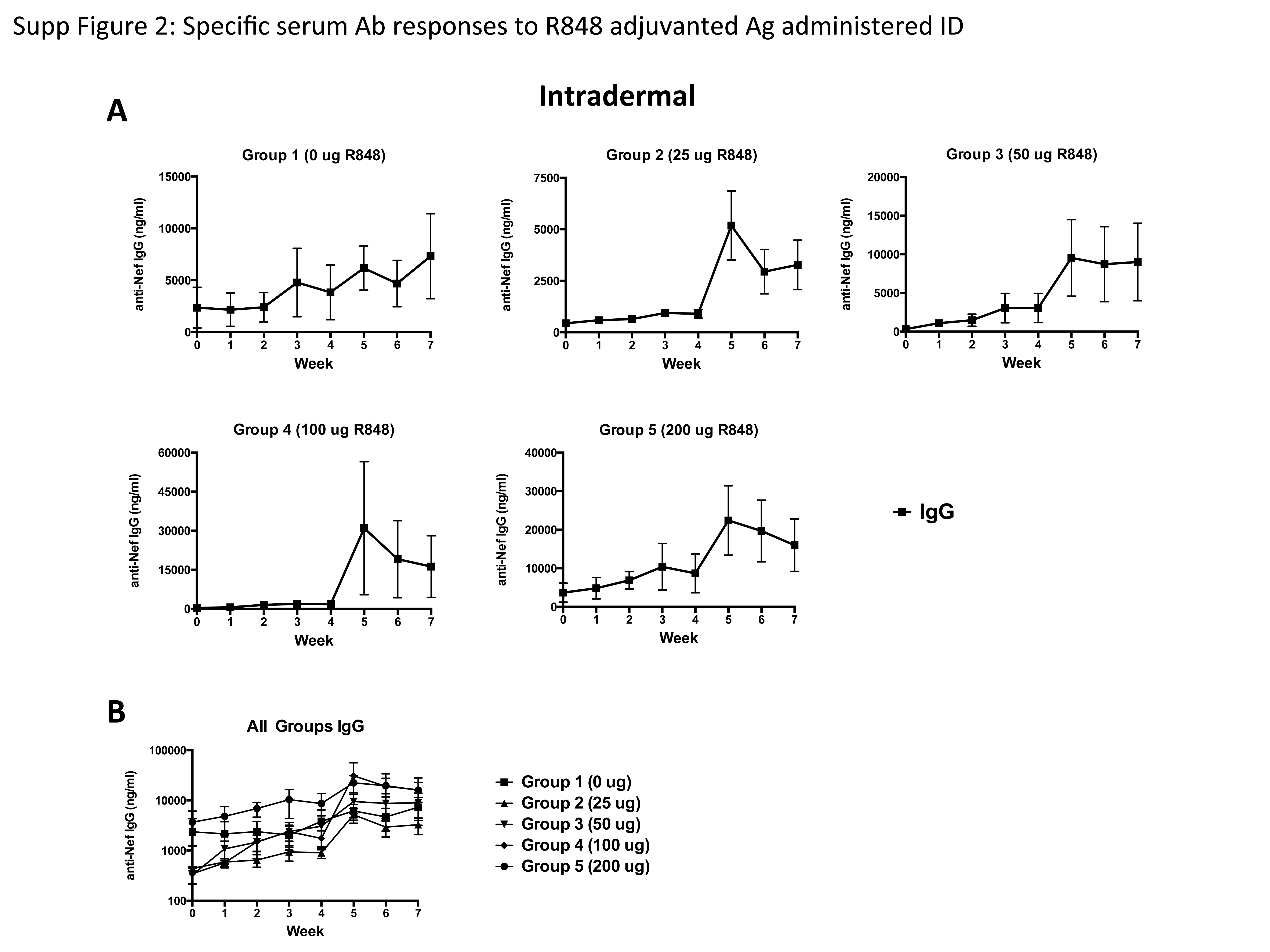

Supplement: S2 Fig — The TLR 7/8 adjuvant R848 administered via the ID route significantly augments antigen-specific serum IgG responses to the Nef HIV viral antigen. A) Amounts of R848 ranging from 200 μg down to 25 μg were formulated with 50 μg Nef and injected ID into the skin at the back of the ear. These pigs exhibited very high anti-Nef IgA antibody background, probably due to cross-reactivity to an endogenous porcine retrovirus. B) Shows the Nef antigen-specific serum IgG responses of all adjuvanted groups compared to the unadjuvanted control. (TIF) [file pone.0148984.s002.tif]

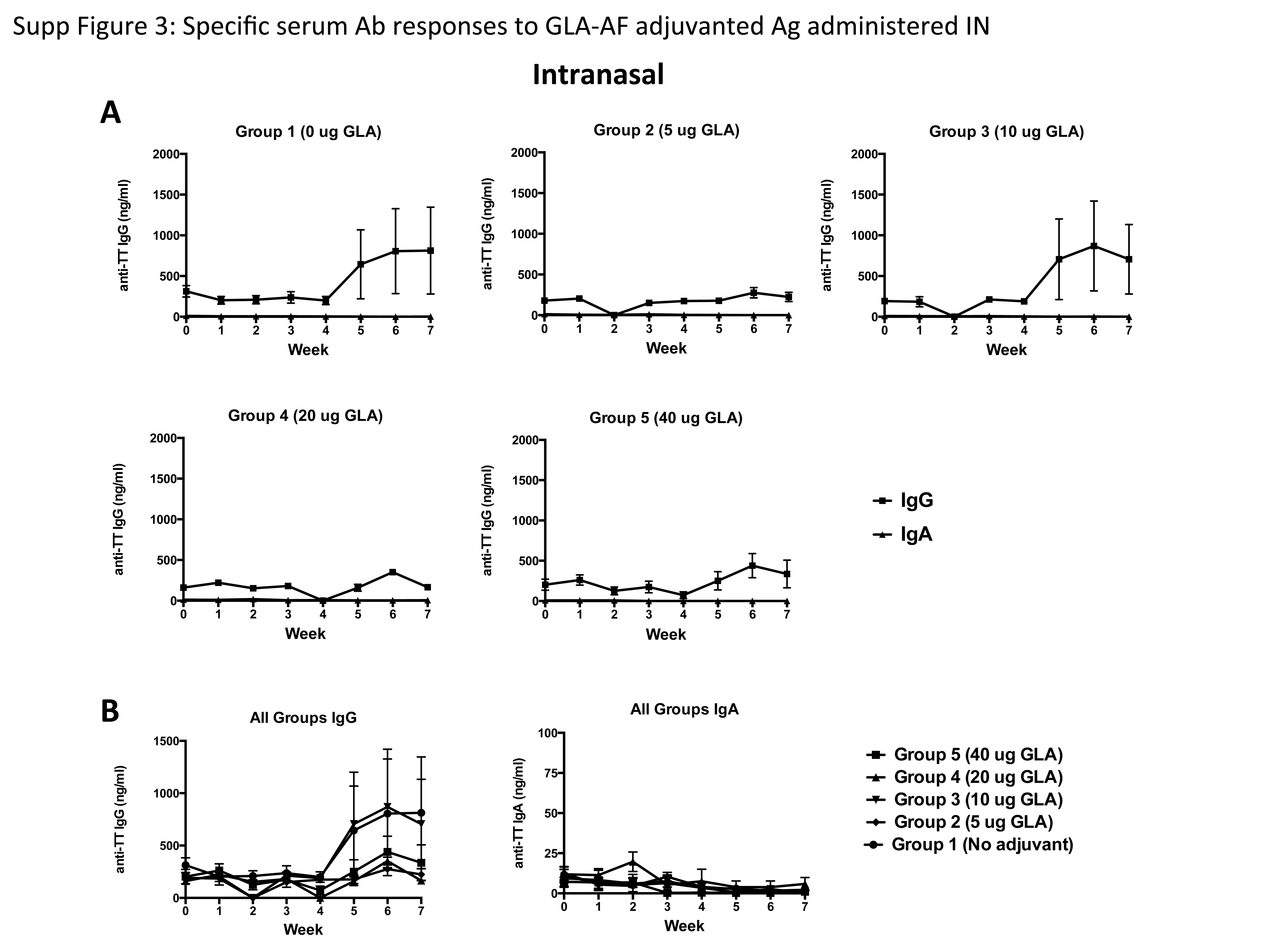

Supplement: S3 Fig — The TLR 4 adjuvant GLA-AF administered via the IN route weakly augments antigen-specific serum IgG but does not enhance IgA responses to the TT (Tetanus Toxoid Fragment c) vaccine antigen. A) Amounts of GLA-AF ranging from 40 μg down to 5 μg were formulated with 50 μg TT and administered directly into the pig nares. B) The antigen-specific serum IgG and IgA responses of all adjuvanted groups compared to the unadjuvanted control. (TIF) [file pone.0148984.s003.tif]

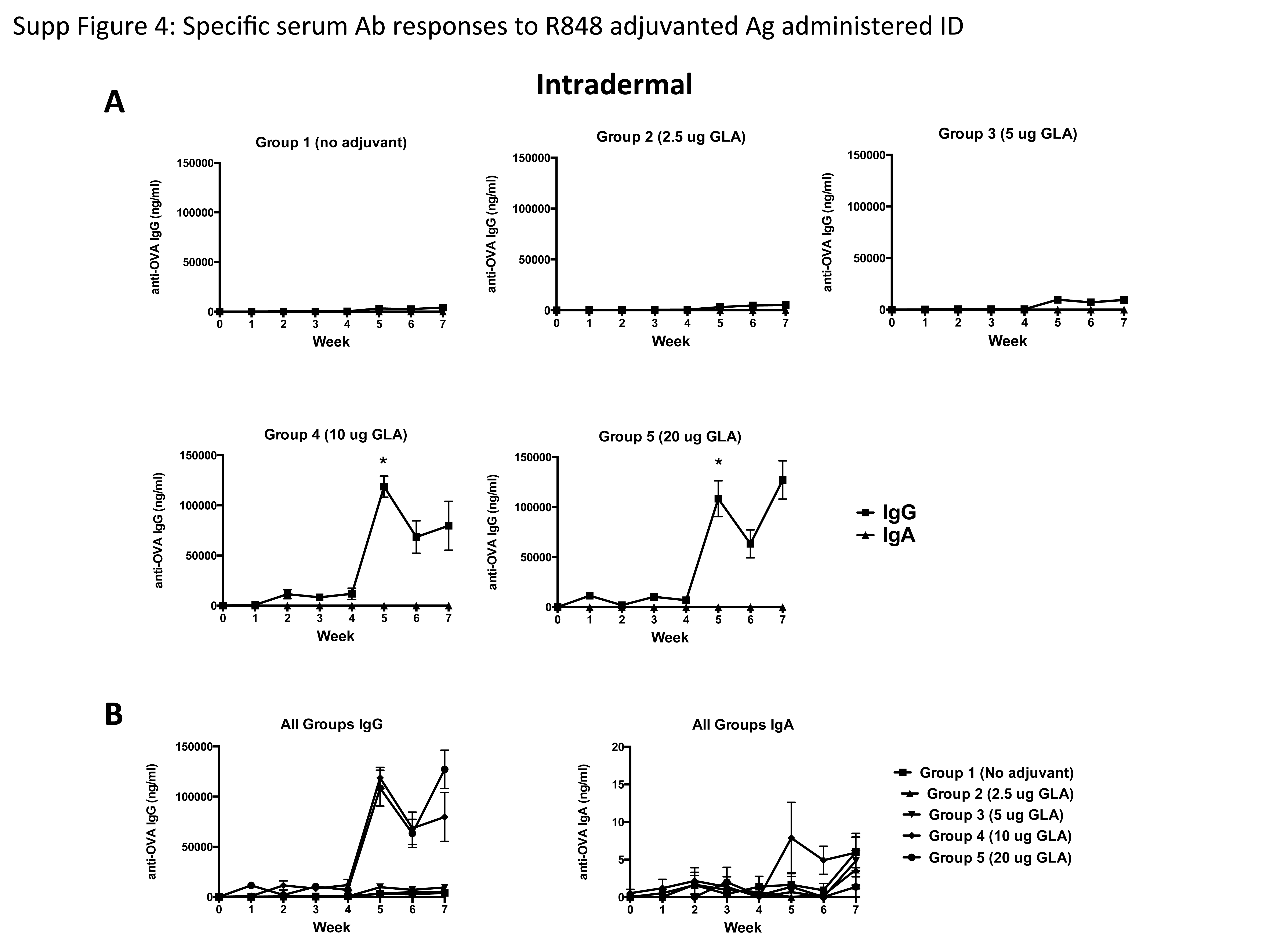

Supplement: S4 Fig — The TLR 4 adjuvant GLA-AF administered via the ID route strongly augments antigen-specific serum IgG but does not enhance IgA responses to the OVA (Ovalbumin) vaccine antigen. A) Amounts of GLA-AF ranging from 20 μg down to 2.5 μg were formulated with 50 μg OVA and injected ID into the skin at the back of the ear, significance is shown at one week after a boost IN inoculation, significance is shown at one week after a boost IN inoculation (*p = 0.0286; 10 and 20 μg). B) Shows the antigen-specific serum IgG and IgA responses of all adjuvanted groups compared to the unadjuvanted control. (TIF) [file pone.0148984.s004.tif]

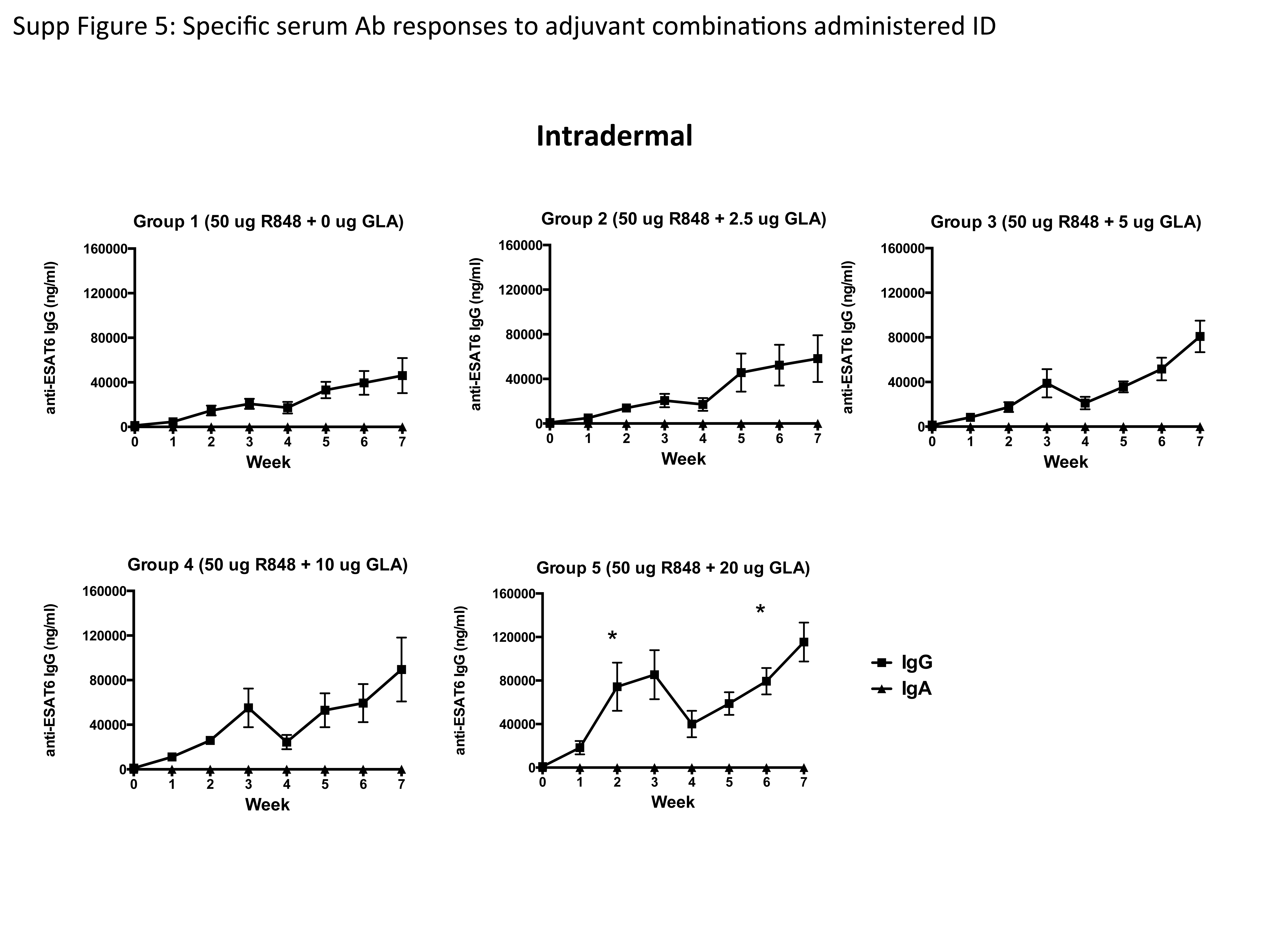

Supplement: S5 Fig — A fixed amount of TLR 7/8 R848 adjuvant was titrated against increasing quantities of co-formulated TLR 4 agonist GLA-AF and administered ID to the skin on the back of the ear. The combination of 50 μg R848 and 20 μg GLA-AF demonstrated significant augmentation of the ESAT-6 (Early secreted antigen target-6 from Mycobacterium tuberculosis) antigen-specific IgG response over that of ESAT-6 delivered with only R848 at both 3 weeks post the primary injection and three weeks after a boost vaccination (*p = 0.033). (TIF) [file pone.0148984.s005.tif]

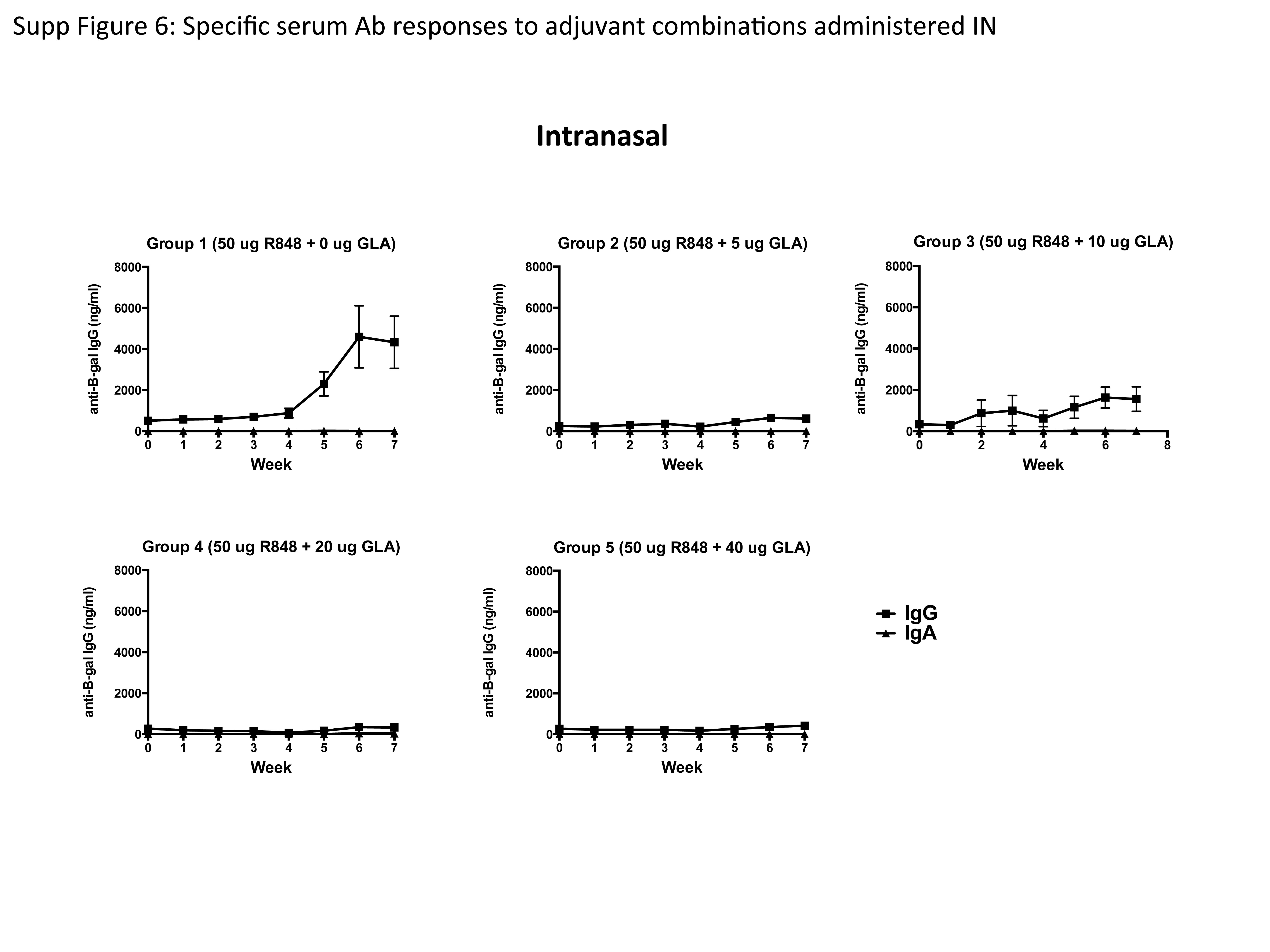

Supplement: S6 Fig — A fixed amount of TLR 7/8 R848 adjuvant was titrated against increasing quantities of co-formulated TLR 4 agonist GLA-AF and administered IN directly into the pig nares. The combination of 50 μg R848 and any quantity of GLA-AF completely ablated the R848 generated response to β-Gal. (TIF) [file pone.0148984.s006.tif]

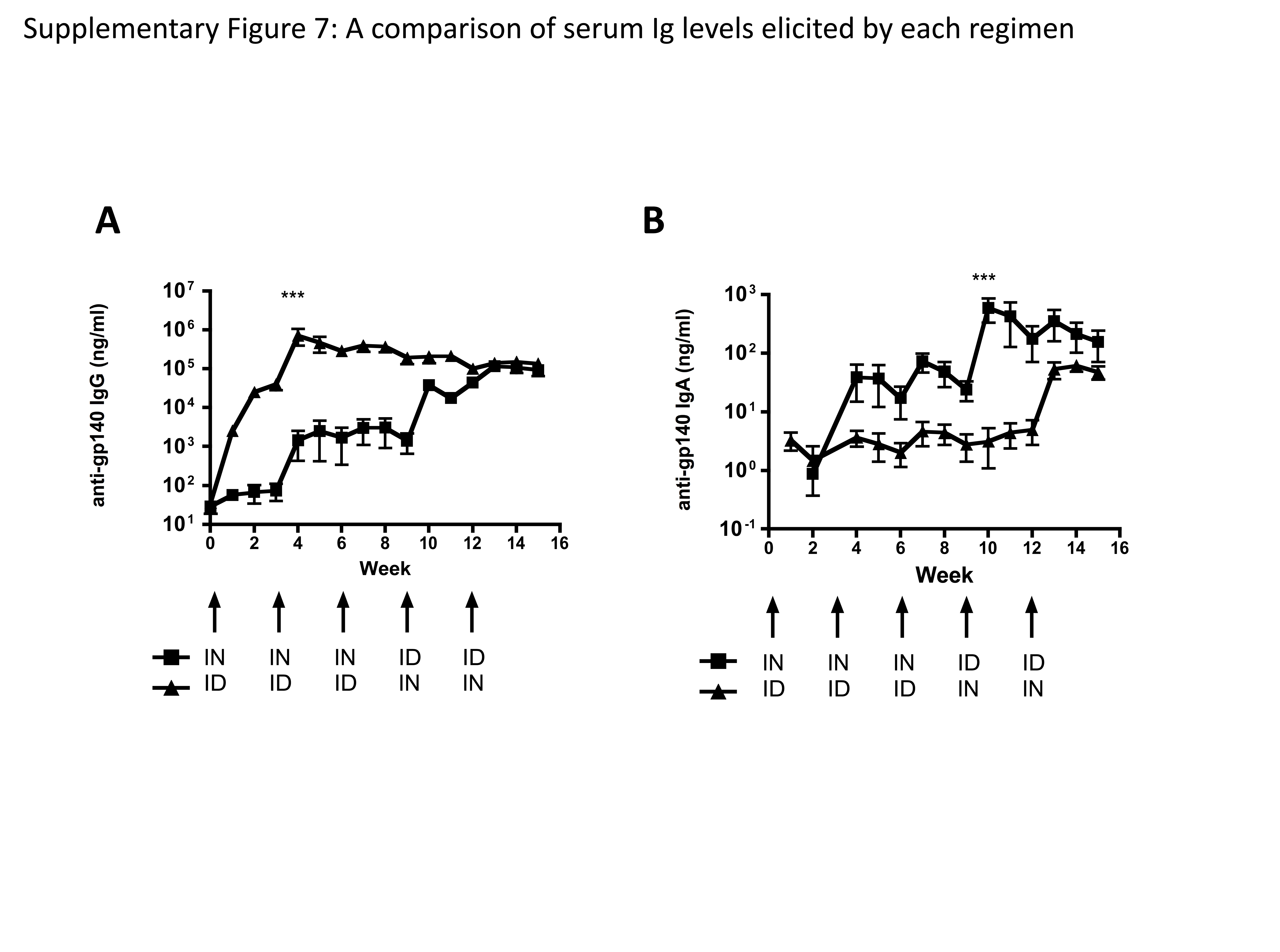

Supplement: S7 Fig — A) Adjuvanted ID injections provide an early enhancement over the IN route (*** p = 0.0006; Week 4) which was lost by the end of the regimen. B) Serum IgA levels are statistically increased in the IN vaccinated pigs (***p = 0.0006; Week 10) over the ID primed animals but this difference was lost at the end of the vaccination schedule. (TIF) [file pone.0148984.s007.tif]

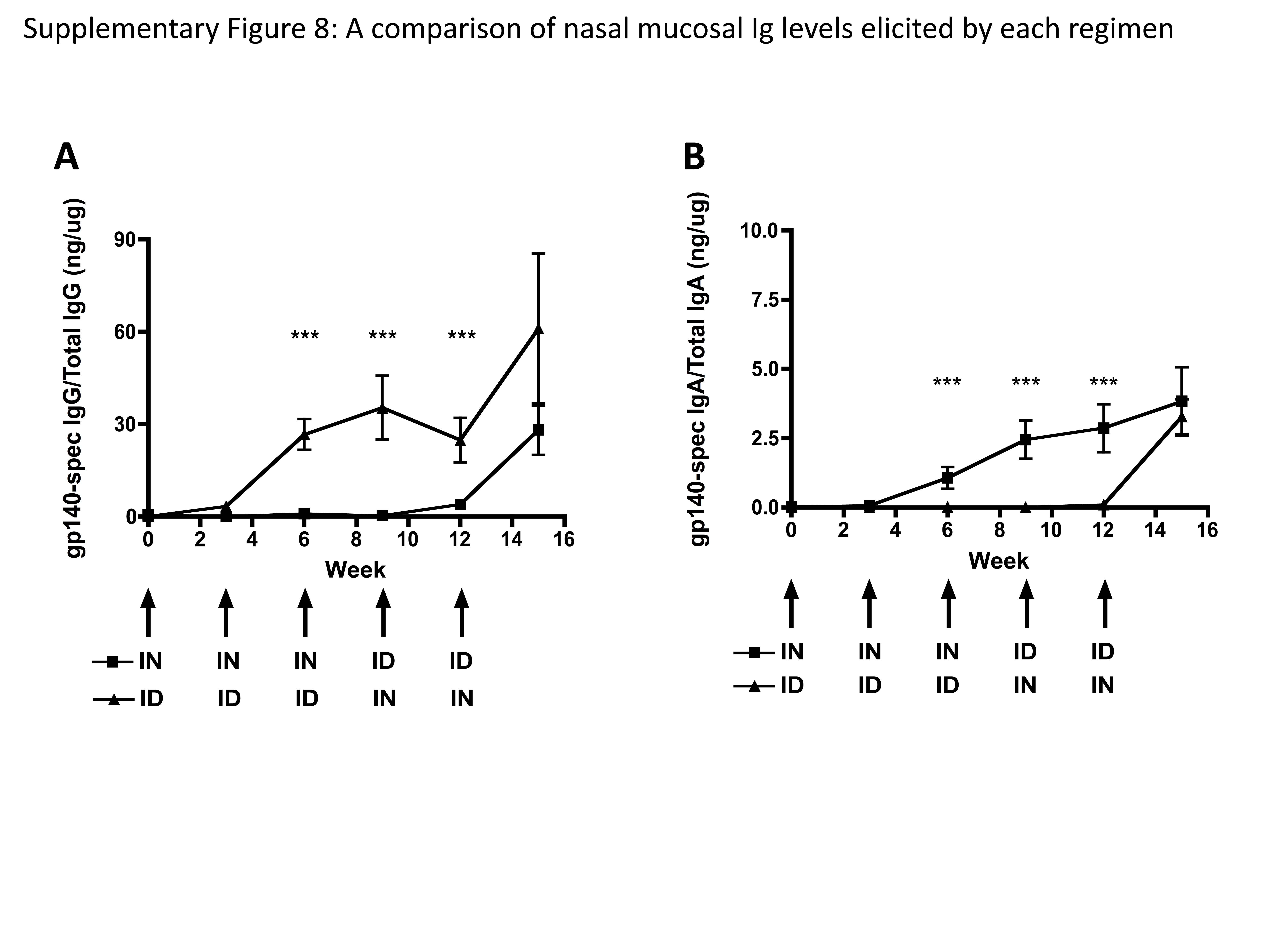

Supplement: S8 Fig — A) Adjuvanted ID injections provided an early and statistically significant enhancement over the IN route (***p = 0.0006; Weeks 6, 9 and 12) which was lost by the end of the regimen. B) Nasal mucosal IgA levels are statistically increased in the IN vaccinated pigs (***p = 0.0006; Weeks 6, 9 and 12) over the ID primed animals but this difference was also lost at the end of the vaccination schedule. (TIF) [file pone.0148984.s008.tif]

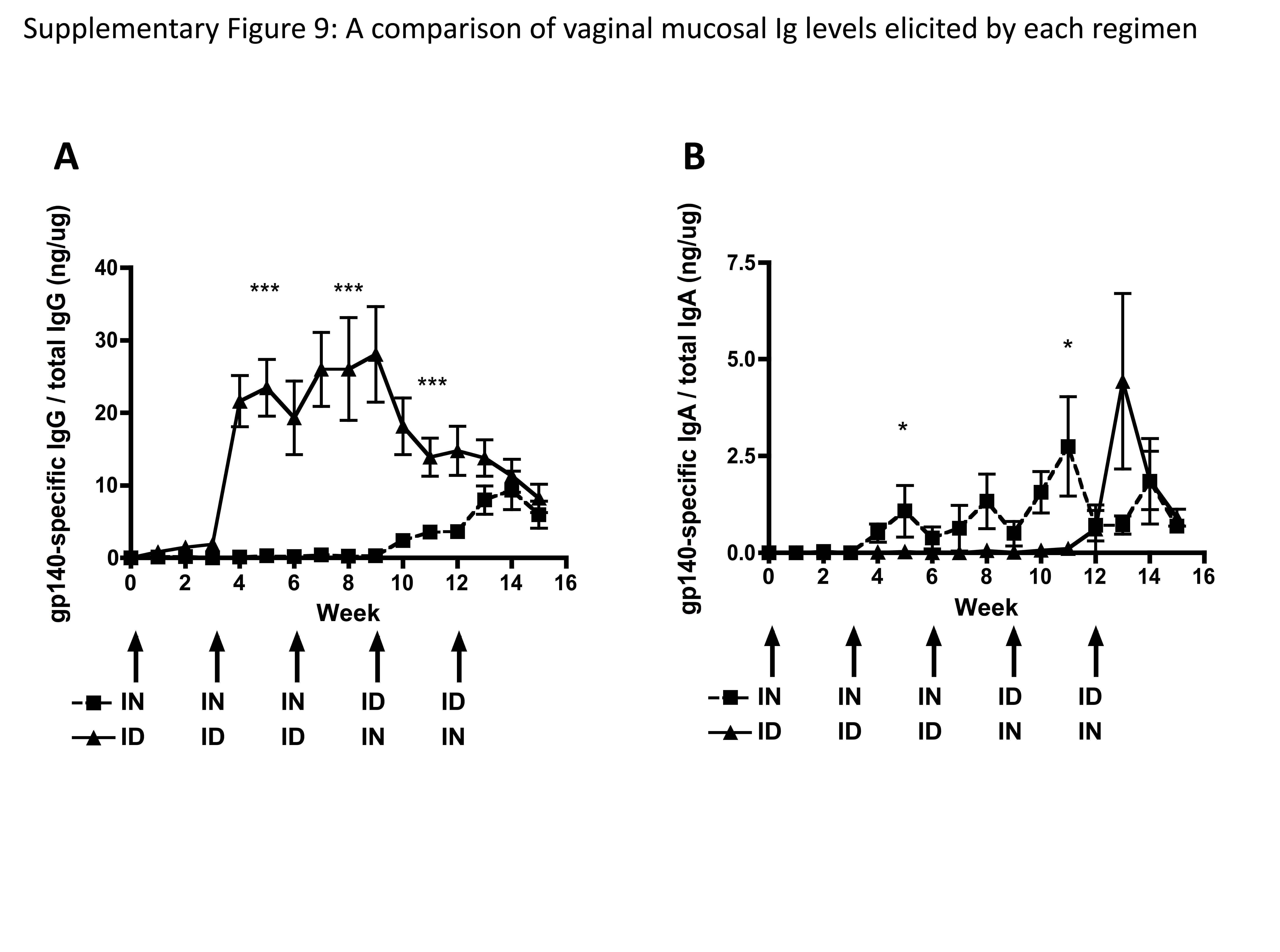

Supplement: S9 Fig — A) Adjuvanted ID injections provided an early and statistically significant enhancement over the IN route (***p = 0.0003; Weeks 5, 8 and 13) which was lost by the end of the regimen. B) Vaginal mucosal IgA levels are statistically increased in the IN vaccinated pigs (*p = 0.0256; Week 5, p = 0.0210; Week 11) over the ID primed animals but this difference was lost at the end of the vaccination schedule. (TIF) [file pone.0148984.s009.tif]
